# Prosocial Reward Learning in Children and Adolescents

**DOI:** 10.3389/fpsyg.2016.01539

**Published:** 2016-10-05

**Authors:** Youngbin Kwak, Scott A. Huettel

**Affiliations:** ^1^Department of Psychological and Brain Sciences, University of MassachusettsAmherst, MA, USA; ^2^Center for Cognitive Neuroscience, Duke UniversityDurham, NC, USA; ^3^Duke Center for Interdisciplinary Decision Sciences, Duke UniversityDurham, NC, USA; ^4^Department of Psychology and Neuroscience, Duke UniversityDurham, NC, USA

**Keywords:** social decision making, adolescence, reward, reinforcement learning, prosociality

## Abstract

Adolescence is a period of increased sensitivity to social contexts. To evaluate how social context sensitivity changes over development—and influences reward learning—we investigated how children and adolescents perceive and integrate rewards for oneself and others during a dynamic risky decision-making task. Children and adolescents (*N* = 75, 8–16 years) performed the Social Gambling Task (SGT, Kwak et al., 2014) and completed a set of questionnaires measuring other-regarding behavior. In the SGT, participants choose amongst four card decks that have different payout structures for oneself and for a charity. We examined patterns of choices, overall decision strategies, and how reward outcomes led to trial-by-trial adjustments in behavior, as estimated using a reinforcement-learning model. Performance of children and adolescents was compared to data from a previously collected sample of adults (*N* = 102) performing the identical task. We found that that children/adolescents were not only more sensitive to rewards directed to the charity than self but also showed greater prosocial tendencies on independent measures of other-regarding behavior. Children and adolescents also showed less use of a strategy that prioritizes rewards for self at the expense of rewards for others. These results support the conclusion that, compared to adults, children and adolescents show greater sensitivity to outcomes for others when making decisions and learning about potential rewards.

## Introduction

Adolescence is commonly characterized as a period of poor decision making manifested in risky, irrational, and often self-destructive choices (Blakemore and Robbins, 2012). Yet, adolescence is also a period of heightened sensitivity to social contexts (Nelson et al., 2005; Blakemore, 2010; Burnett et al., 2011; Crone and Dahl, 2012), consistent with the importance of acquiring skills necessary for independent contributions to society. To achieve this end, adolescents are motivated to build social networks amongst their peers and to take actions that strengthen those social networks (Crone and Dahl, 2012). With the increase in motivation for social belonging, social cognitive skills also peak in adolescence as reported by studies of mentalizing, meta-cognition, perspective taking, and social reasoning (Blakemore, 2008; Burnett et al., 2011). The development of these essential social cognitive skills allows adolescents to move beyond self-oriented thoughts and behaviors and to start engaging in socially-oriented behaviors (Eiesnberg and Fabes, 1998).

Adolescents' increase in sensitivity to social values has been repeatedly shown by social decision making studies using one-shot interactive games such as the ultimatum game, dictator game, and trust game (Harbaugh and Krause, 2000; Sutter, 2007; Güroğlu et al., 2009; Almås et al., 2010; van den Bos et al., 2010, 2011, 2014; Overgaauw et al., 2012; Steinbeis et al., 2012; Fett et al., 2014; Güroğlu et al., 2014; Steinmann et al., 2014). These studies show that the basic mechanisms underlying one's prosocial tendencies such as considerations for fairness, altruism, trust, and reciprocity continue to develop from childhood through adolescence (Güroğlu et al., 2009; Crone and Dahl, 2012). During this period, the other-oriented prosocial tendencies can become more dominant than self-oriented thoughts (Crone and Dahl, 2012). There have also been studies looking at the developmental trajectory of prosocial behaviors across adolescence using self-report measures (Eisenberg et al., 1995, 2002, 2005; Eiesnberg and Fabes, 1998; Killen and Turiel, 1998; Carlo et al., 1999, 2007; Fabes et al., 1999; Wentzel et al., 2007; Luengo Kanacri et al., 2013). These studies show mixed findings of consistent increase in prosocial tendencies or a quadratic change across age showing decrease in prosociality until mid-adolescence and then an increase toward late adolescence. Increased sensitivity to social contexts is in line with the developmental changes in sensitivity to “hot” (high affective arousal state) contexts, which peaks around adolescence (Boyer and Byrnes, 2009).

While results from one-shot interactive games and from self-reports have provided important insights about the development of social preferences, both sorts of measures have inherent limitations. Both involve explicit, direct probes of prosociality, which may increase their susceptibility to experimenter bias particularly in younger samples (Bryan and London, 1970; Zarbatany et al., 1985; Innocenti and Pazienza, 2006). One-shot interactive games also provide only very limited data—often a single choice per participant—which precludes analysis of learning effects. Everyday social decisions, in contrast, are often made in a dynamic environment that requires adjusting our behavior based on the past experience (e.g., learning from past rewards; Erdem and Keane, 1996).

In a recent study, we developed a Social Gambling Task (SGT) that measures how learning of rewards unfolds under a social environment (Kwak et al., 2014). Specifically, the task incorporates non-social and social rewards into an interactive and dynamic card game. Based upon the well-studied Iowa Gambling Task (Bechara et al., 1994), the SGT contains four decks that each have a different relative distribution of payoffs for oneself and for a desirable charity. We used charitable donations as a prototypic method for eliciting social preferences as they reliably engage processes associated with social cognition (Tankersley et al., 2007; Zaki and Mitchell, 2011) and with reward evaluation (Moll et al., 2006; Harbaugh et al., 2007). We also employed a reinforcement-learning model to understand the course of reward learning throughout the task. Converging evidence from choices and modeling results showed that people readily learn about others' reward contingencies, just as they do for their own rewards, although there were significant individual differences in self vs. charity reward learning reflecting variability in the expressions of prosocial behaviors like altruism, fairness, and trust (Fehr and Schmidt, 2003; Kishida et al., 2010; Murphy et al., 2011). More specifically, greater relative valuation for social rewards compared to self rewards as indexed by a reward weighting parameter of the model was associated with individual differences in self-reported prosocial tendencies (Kwak et al., 2014). Thus, the SGT provides a well-defined laboratory index of prosociality that not only shares features of more natural decision tasks (e.g., implicit learning, dynamic information acquisition) but also relates to independent measures of other-regarding behavior.

In the current study, we investigated how children in the age of pre-adolescence and adolescence (8–16 years) perceive and integrate rewards of both self and others in the SGT. To assess how reward outcomes influenced choice behavior, we applied a reinforcement learning model and compared both choices and model estimates between children/adolescents and adults. Our previous study demonstrated that the SGT gives a measure of how an individual balances rewards of oneself versus others. Based on the current literature on the development of social values, we hypothesized that children and adolescents (compared to adults) will exhibit increased sensitivity toward rewards directed to others.

## Materials and methods

### Participants

A total of 75 pre-adolescent children and adolescents (32 males and 43 females, age range = 8–16 years, *M* = 12 years, *SD* = 3 years) participated in this study. Performance was compared to data from young adults in a previously collected and reported study (*N* = 102; 44 men, 58 women; age range = 18–36 years, *M* = 23 years, *SD* = 4 years) (Kwak et al., 2014). Children and adolescents were recruited from local private schools in greater Durham, NC; these schools' demographics (e.g., socioeconomic status) are largely similar to that of the young adult population from which our prior adult sample was drawn. Children and adolescents were tested in our behavioral testing laboratory on the Duke campus; this testing environment was identical to that previously used for the adult participants. To examine the effect of age, we additionally performed correlational analyses using age as a covariate within our child/adolescent age group. All children and adolescents provided parent consent and child assent under a protocol approved by the Duke University Institutional Review Board.

### Procedure

At the outset of the experiment, participants viewed instructions on the study paradigm. Then, they read a brief introduction to a charity foundation to which earnings would be donated. As a charity with broad appeal to the age range studied, we used the “Make a Wish!” foundation, which helps young children with severe illness accomplish their dreams. The introduction to the foundation included example stories of children whose wishes come true (e.g., becoming a firefighter) with the help of the foundation. Participants then performed the Social Gambling Task (SGT) described below. After completion of the task, participants filled out the Helping Orientation Questionnaire (HOQ), an index of prosocial personality (Romer et al., 1986) as well as several questions regarding the charity foundation. Details about the questionnaires are in the Supplementary Materials. At the end of each experimental session, the experimenter paid the participant and made a donation to the charity (both ranging from $10 to $16) according to their choices. The participants were explicitly told that the amount of money they won for the charity in this task (ranging from $10 to $16) will be donated to the foundation.

### Social gambling task (SGT)

**Figure 1:**
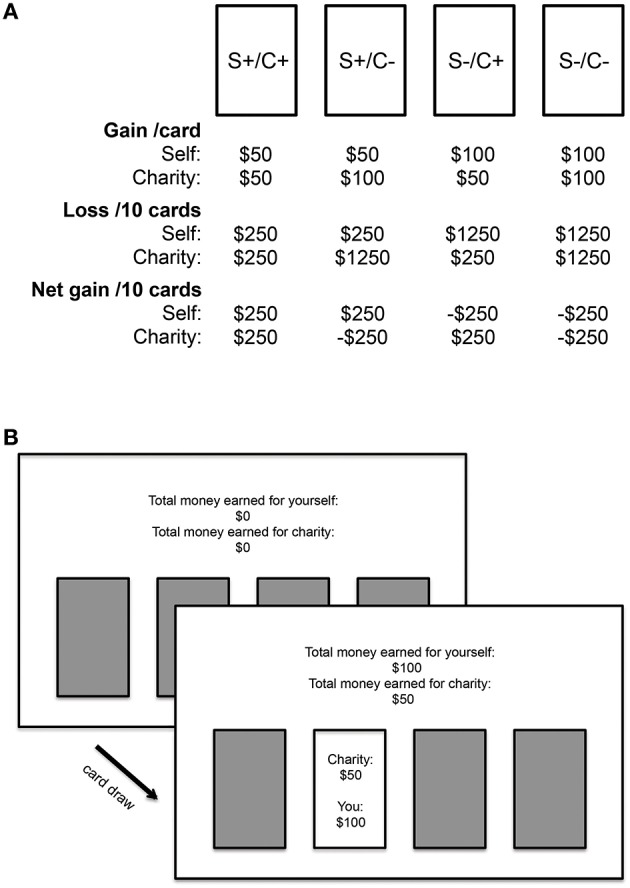
**Payout structure for each card deck of the Social Gambling Task (SGT)**. S = self, C = charity, + = gain deck, − = loss deck. For the gain decks, each card draw always gave $50 but in some trials it was also associated with losses ranging between $25 and $75 (i.e., loss of $25, $50, or $75). Each card draw only displayed the outcome combining the gain and loss, which ranged between −$25 to $50. Every 10 trials had 5 loss trials with a total loss of $250, thus resulting in a net gain of $250. For the loss decks, each card draw always gave $100 with some trials giving losses ranging between $150 and $350 (i.e., loss of $150, $200, $250, $300, or $350). The outcome displayed ranged between −$250 to $100. Every 10 trials had 5 loss trials with a total loss of $1250, thus resulting in a net loss of $250. **(A)** An example screen display of a trial in SGT **(B)**. Figure adapted from Kwak et al. (2014).

SGT modifies the basic structure of the Iowa gambling task (IGT; Bechara et al., 1994), such that each card deck is associated not only with monetary outcomes for the player, but also monetary outcomes for the charity. The four decks had differing payoffs for self versus charity in a 2 × 2 fully orthogonal design (S+/C+, S+/C−, S−/C+, S−/C−; S = self, C = charity, + = gain deck, − = loss deck); see Figure 1 for details on card payout structure. For the display of outcomes of gain decks (Decks S+/C+ and S+/C−, for self; Decks S+/C+ and S−/C+ for charity), each card draw gave $50 (for either self or charity), but in some trials it was also associated with losses ranging between $25 and $75 (i.e., loss of either $25, $50, or $75). Each card draw displayed one outcome value (i.e., either −$25, $0, $25, or $50), which was the net outcome combining the gain and loss. Every 10 trials had 5 loss trials with a total loss of $250, thus resulting in an accumulated net gain of $250. For the display of outcomes of loss decks (Decks S−/C+ and S−/C−, for self; Decks S+/C− and S−/C−, for charity), each card draw gave $100 (for either self or charity) with some trials giving losses ranging between $150 and $350 (i.e., loss of either $150, $200, $250, $300, or $350), resulting in one net outcomes value (i.e., either −$250, −$200, −$150, −$100, −$50, or $100). Every 10 trials had 5 loss trials with a total loss of $1250, thus resulting in an accumulated net loss of $250. The four card decks were horizontally displayed in the center of the screen. After the player chose a deck, two separate payouts pertaining to self and to charity appeared. After a total of 100 selections, the task automatically stopped without warning; participants did not know how many trials the task comprised. The total accumulated amount won for self and for charity was shown on top of the screen on every trial. The specific instructions we gave to the participants regarding the task were described in the Supplementary Materials.

### Reinforcement learning model

We applied a reinforcement learning model to characterize the reward-learning process (Kwak et al., 2014). Decisions were assumed to be made based on action values of each card deck, calculated in response to the history of received rewards of both types. Specifically, the value (V_ij_) of deck *i* on a given trial *j* is defined as:

(1) $$\begin{array}{l} {\text{V}}_{\text{ij}}=\alpha {\text{Q}}_{\text{Sij}}+\left(1-\alpha \right){\text{Q}}_{\text{Cij}} \end{array}$$

where (Q_Sij_) and (Q_Cij_) are the estimated rewards to self and charity, for deck *i* on trial *j*. The parameter α ranges between completely self-interested (α = 1) and completely charity-interested valuation (α = 0), as fit individually for each subject based on choice patterns. Q_Sij_ and Q_Cij_ are updated each time the particular deck is chosen.

(2) $$\begin{array}{l} {\text{Q}}_{\text{Sij}}={\text{Q}}_{\text{Si}\left(\text{j}-1\right)}+\lambda_{\text{S}}\left({\text{R}}_{\text{Sj}}-{\text{Q}}_{\text{Si}\left(\text{j}-1\right)}\right) \end{array}$$

(3) $$\begin{array}{l} {\text{Q}}_{\text{Cij}}={\text{Q}}_{\text{Ci}\left(\text{j}-1\right)}+\lambda_{\text{C}}\left({\text{R}}_{\text{Cj}}-{\text{Q}}_{\text{Ci}\left(\text{j}-1\right)}\right) \end{array}$$

R_S_ and R_C_ are the observed reward outcomes for self and charity on the current trial *j*, λ_S_, and λ_C_ are learning rates, and (R_j_–Q_j−1_) are the reward prediction errors for each reward type.

Our model links option values (*V*_*ij*_) with choice probabilities via the softmax rule (Sutton and Barto, 1998).

(4) $$\begin{array}{l} P\left(i|\beta ,V_{ij}\right)=\frac{e^{\beta V_{ij}}}{\sum_{j}e^{\beta V_{ij}}} \end{array}$$

Here, P is the probability of choosing a particular deck *i* on a given trial *j* and β is a so-called “greediness” parameter with β = ∞ corresponding to perfectly greedy (exploitive) choice behavior.

Option values *V*_*ij*_ are weighted sums of separately learned self and charity values for each deck, with the proportion controlled by a parameter α. We fit models using a maximum log likelihood method implemented with MATLAB's constrained optimization routines (i.e., fmincon). For each subject, we fit β, α, and two learning rates that captured responses to self and charity rewards separately.

### Data analysis

SPSS Statistics 20.0.0 statistical software (SPSS Inc., Chicago, IL) was used for all statistical analyses. The statistical threshold for all analyses was a *p* value of 0.05. When computing tests for repeated measures data, the Huynh–Feldt epsilon (Huynh and Feldt, 1970) was used to determine whether data met the assumption of sphericity (Σ > 0.75). In cases where the sphericity assumption was not met, the F statistic was evaluated for significance using the Huynh–Feldt adjusted degrees of freedom. The reinforcement learning model was implemented using MATLAB (The Mathworks, Natick, MA).

## Results

### Social gambling task

Learning of the reward contingencies in the self and charity domain was quantified as the Learning Index (LI): the relative proportion of choices from gain decks versus loss decks. Specifically, learning indices for self (LI_self_) and for charity (LI_charity_) were independently calculated as follows:

(5) $$\begin{array}{l} {\text{LI}}_{\text{self}}=(\#\text{ of cards drawn from S }+\;/\text{C}+\text{ and S }+\;/\text{C}-\\ \text{ decks})-(\#\text{ of cards drawn from S }-\;/\text{C }+\text{ and S }-\;/\\ \text{ C }-\text{ decks}) \end{array}$$

(6) $$\begin{array}{l} {\text{LI}}_{\text{charity}}=(\#\text{ of cards drawn from S }+\;/\text{C }+\text{ and S }-/\text{C}+\\ \text{ decks})-(\#\text{ of cards drawn from S }+\;/\text{C}-\text{ and S }-\;/\\ \text{ C }-\text{ decks}). \end{array}$$

**Figure 2:**
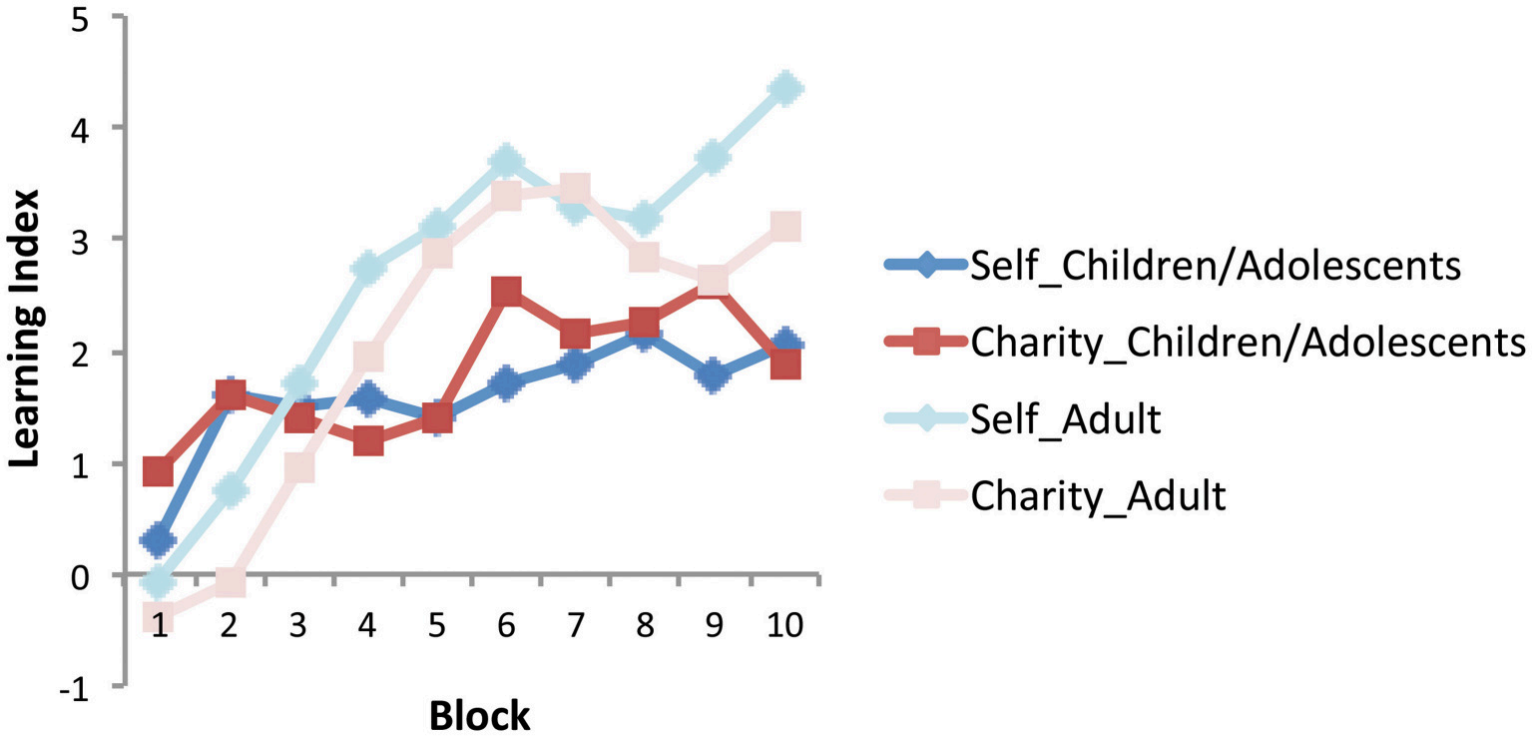
**Learning index across the 10 blocks (10 card draws each) for self and charity domain in children/adolescents and adults**. The results from adults were previously reported in Kwak et al. (2014). Learning index was independently calculated in the self and charity domain as the difference in the number of gain decks and loss decks chosen.

To determine how learning emerged across time for self and charity in the children/adolescent and adults, we looked at the changes in Learning Indices (LI_self_ and LI_charity_) across 10 blocks of 10 trials each. A repeated measures ANOVA with domain (LI_self_ vs. LI_charity_) by block (block 1–10) as within-subject factors and age group (children/adolescents vs. adults) as a between-subject factor showed a significant main effect of block [*F*_(6.34, 1109.01)_ = 17.01, *p* < 0.0001] and an age-group-by-block interaction [*F*_(6.34, 1109.01)_ = 5.55, *p* < 0.0001] (Figure 2). *Post hoc* testing revealed that the linear [*F*_(1, 175)_ = 48.31, *p* < 0.0001] and quadratic [*F*_(1, 175)_ = 16.52, *p* < 0.0001] contrasts for block were significant and the interaction with age group using these two contrasts were also significant [linear: *F*_(1, 175)_ = 11.75, *p* = 0.001, quadratic: *F*_(1, 175)_ = 5.81, *p* = 0.017]. These results indicate that both the children/adolescents and adults learned the reward contingencies over the task and that the two age groups showed different rates in improvements over time, with adults showing a steeper improvement over time. No other main effects or interaction effects were found. No main effects or interaction effects were found when using the total learning index across all trials.

We also compared the choice strategies used by the two age groups. Specifically we looked at how people adapted their choice behavior following positive (i.e., win) or negative (i.e., lose) outcomes. We computed the proportion of choices that were consistent with the following four different strategies: same choice of card deck as the previous trial when the outcome of the previous trial was self win and charity win (WW), self win and charity lose (WL), self lose and charity win (LW), and self lose, and charity lose (LL).

We first examined whether subjects' strategies predicted overall learning by looking at the correlation between LI difference (LI_charity_–LI_self_) measure and each of the strategies. A significant negative correlation (with Bonferroni correction for 4 different correlation analyses, *p* < 0.05/4) was found only with WL strategy (*r* = −0.24, *p* = 0.001) suggesting that a greater use of this strategy is associated with less prosocial and more selfish reward learning across the entire task.

**Figure 3:**
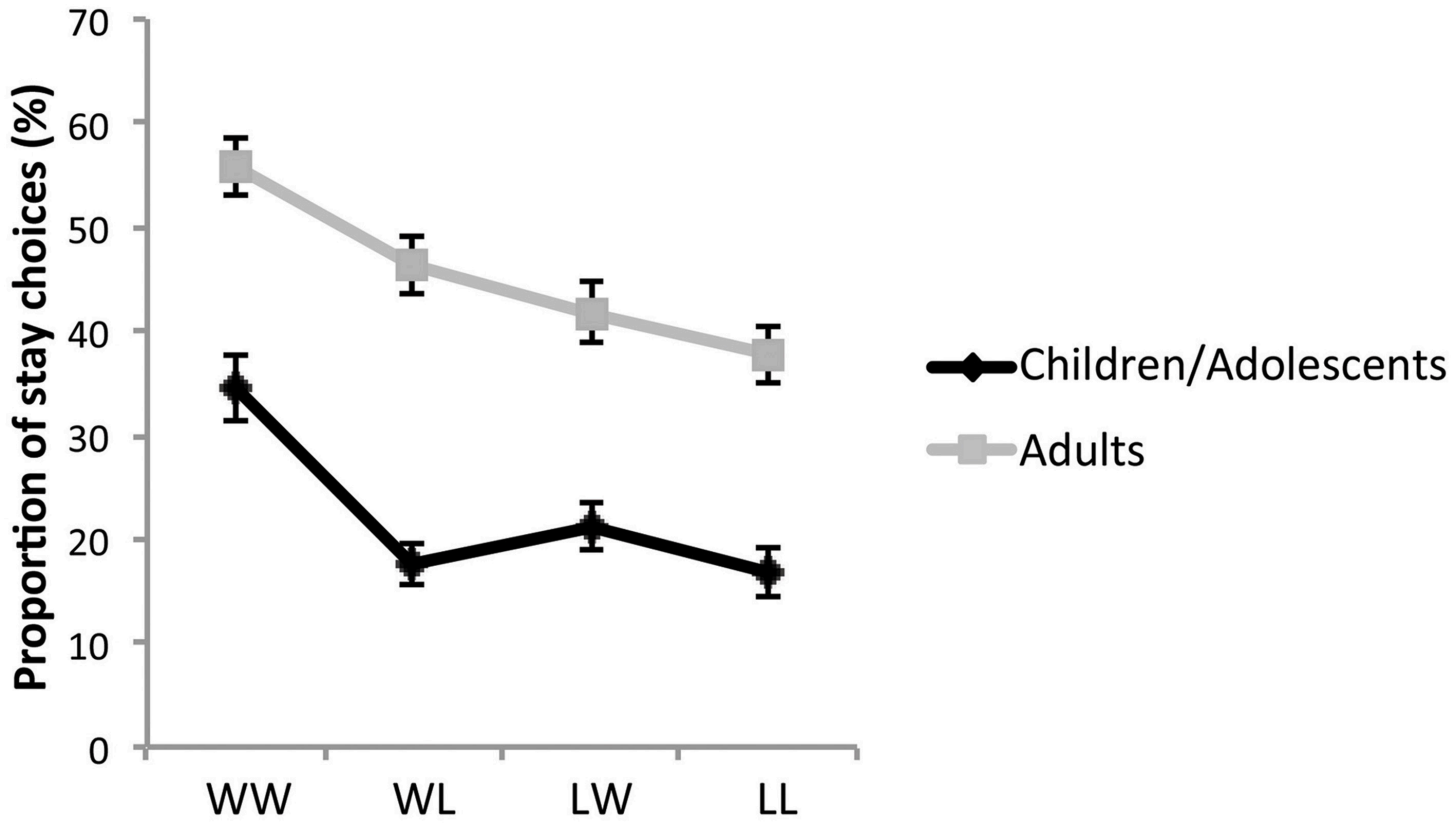
**The proportion of choices where the same deck was chosen from previous trial (i.e., proportion of stay choices) across four different outcome scenarios of self and charity in children/adolescents and adults**. WW refers to the case in which there were positive outcomes for both self and charity, WL refers to a positive outcome for self and a negative outcome for charity, LW refers to a negative outcome for self and a positive outcome for charity and LL refers to negative outcomes for both self and charity. Error bars indicate standard error.

Next, we compared the use of the four strategies across age groups using a repeated- measures ANOVA with age group as a between-subject factor and strategy type as a within-subject factor. We found a main effect of strategy type [*F*_(3, 525)_ = 45.15, *p* < 0.0001], age group [*F*_(1, 175)_ = 47.10, *p* < 0.0001] and an age-group-by-strategy-type interaction [*F*_(3, 525)_= 2.83, *p* = 0.038] (Figure 3). Children/adolescents were using all of these strategies less frequently than adults. To interpret the interaction effect, the repeated contrast was applied to the factor “strategy type.” Significant interaction with age was found in WW vs. WL [*F*_(1, 175)_ = 6.58, *p* = 0.011] and WL vs. LW [*F*_(1, 175)_ = 7.64, *p* = 0.006] but not in LW vs. LL [*F*_(1, 175)_ = 0.03, *p* = 0.854] contrast. This implies that the age group difference in the use of decision strategies is significantly greater in WL than the other three strategies, with the reliance on this strategy being particularly reduced in children/adolescents compared to adults. To confirm whether the interaction effect was driven by the WL strategy, we ran the same ANOVA model including only the other three strategies. The age-by-strategy-type interaction was no longer significant [*F*_(2, 350)_ = 0.036, *p* = 0.97] indicating that the previous age-by-strategy-type interaction was driven by the WL strategy. These results suggest that compared to adults, children and adolescents show decreased use of a strategy that prioritizes rewards for self at the expense of rewards for others.

To determine how reward learning and strategy use change across development, we looked at the correlation of those measures with age within the children/adolescents sample alone. We found a significant positive correlations (with Bonferroni correction for six different correlations, *p* < 0.05/6) between age and LI_self_ (*r* = 0.41, *p* < 0.0001) and between age and WW strategy (*r* = 0.37, *p* = 0.001) suggesting that learning in self domain and the use of WW strategy increase with age (Supplementary Figure 1). These results indicate that within this age-range of pre-adolescence through adolescence (8–16 years), there already exist important developmental changes in reward learning and strategy use. No other relationships were significant.

In an effort to characterize the behavior of children and adolescents at a population level as compared to adults, we categorized individuals into subgroups based on the absence or presence of evidence showing knowledge of the reward contingencies of the card decks. Presence of knowledge was evaluated according to whether a significantly greater number of choices were made in the gain decks compared to the loss decks throughout the entire experiment (knowledge present: LI > 18, knowledge absent: LI ≤ 18, based on binomial test at *p* < 0.05, one-tailed) (Bakos et al., 2010). This categorization was done in both self and charity domain, an approach that was taken in our previous study (Kwak et al., 2014). This led to the following subgroups in both the children/adolescents and the adults: individuals showing knowledge in both the self and charity (Knowledge All group), individuals showing knowledge only in self but not in charity (Knowledge Self group), individuals showing knowledge only in charity but not in self (Knowledge Charity group), and a group who did not show evidence of knowledge for both self and charity domain (No Knowledge group) (Table 1). Although chi square test did not show significant difference in the proportion of the four subgroups (χ^2^ = 6.92, *p* = 0.074) the proportion of Knowledge Charity group was greater in children and adolescents (14.7%) than in adults (10.7%) whereas the proportion of Knowledge Self group was greater in adults (11.8%) than in children/adolescents (6.7%).

**Table 1:** **Number and proportion of participants in each subgroup**.

	**Knowledge all**	**Knowledge self**	**Knowledge charity**	**No knowledge**
Children/Adolescents (%)	19 (25.3)	5 (6.7)	11 (14.7)	40 (53.3)
Adults (%)	40 (39.2)	12 (11.8)	8 (7.8)	42 (41.2)

We noticed that there was a greater proportion of No Knowledge group in children and adolescents (53.3%) than in adults (41.2%). In order to clarify whether the age group differences are driven by differences in their ability to learn, we conducted our analyses comparing learning indices and decision strategies across age groups, after excluding the No Knowledge group from both the children/adolescents and adults. The results did not change significantly (see Supplementary Materials).

### Reinforcement learning model

We first assessed whether the model predicted choices equally well in children/adolescents and adults. On each trial, we identified the deck with the highest probability of being chosen as the model's prediction (see Methods). In trials in which there were two or three options tied in value, subject's choice of one of these tied values was considered correct. To be conservative, trials with all four options tied were considered as incorrect trials. The model correctly predicted a higher proportion of the choices made by adults (0.49 ± 0.24) than by children and adolescents (0.40 ± 0.18) [*t*_(174.6)_ = 2.74, *p* = 0.007]. Based on our previous study (Kwak et al., 2014), we excluded the No Knowledge group from the reinforcement-learning analyses because those individuals' choice could not be successfully predicted by the model.

We compared the self vs. charity bias (α) and the learning rates for self and charity (λ) between the two age groups (Table 2). Within our model, an α near 1 indicates that subjects chose almost entirely based on rewards to themselves, while near 0 indicates that subjects focused solely on charitable outcomes. Children and adolescents had a marginally lower α [*t*_(94)_ = 1.88, *p* = 0.064]. No significant differences were found in the learning rates. We also explored whether the model parameters correlate with age within the children/adolescents sample. We found a significant positive relationship (with Bonferroni correction for 4 different correlations, *p* < 0.05/4) with percent accurately predicted choices (*r* = 0.46, *p* = 0.005), suggesting that model performance increases with age.

**Table 2:** **Model parameters in the two age groups**.

	**Model performance (proportion correct)**	**Self/Charity reward sensitiviy (α = 1, purely selfish; α = 0, purely charitable)**	**Learning rate self (λs)**	**Learning rate charity (λc)**
Adults (M ± SD)	0.63 ± 0.17	0.51 ± 0.34	0.29 ± 0.36	0.28 ± 0.37
Children/Adolescents (M ± SD)	0.53 ± 0.16	0.38 ± 0.34	0.39 ± 0.39	0.25 ± 0.34

For both adults and the children/adolescents, data from No Knowldege group was excluded from the reinforcement learning model analyses.

### Other-regarding behavior

**Figure 4:**
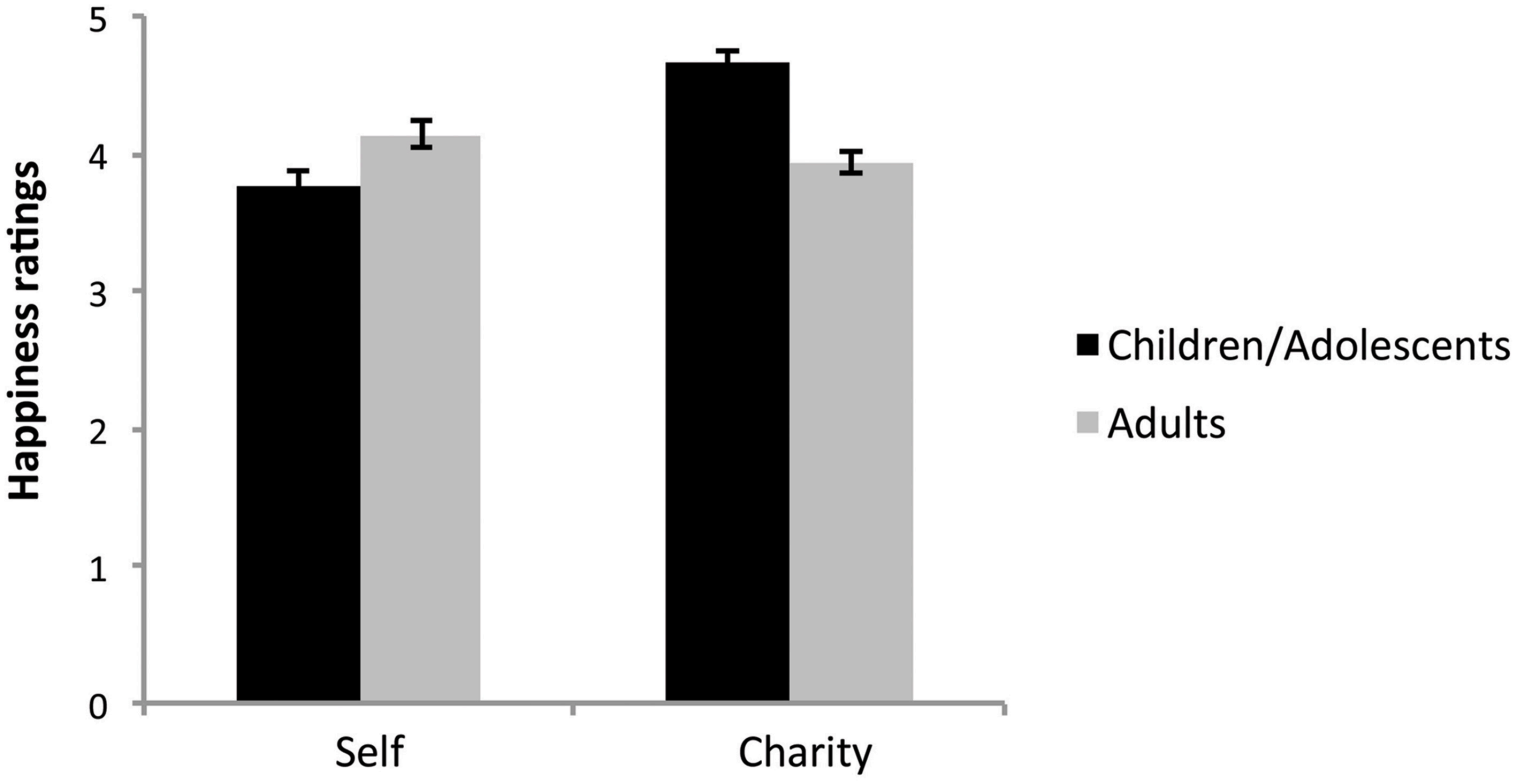
**Happiness rating for winning money for self vs. charity in children/adolescents and adults**. Higher ratings indicate greater happiness reported associated with winning money. Error bars indicate standard error.

To determine how the two age groups differed in terms of their overall other-regarding behavior assessed independently from SGT performance, the altruism and selfishness scores of the Helping Orientation Questionnaire (HOQ) were compared in the two groups. Mean altruism score was significantly higher in children/adolescents (*M* ± *SD*: 11.95 ± 3.56) than in adults (10.11 ± 3.72) [*t*_(160)_ = −3.09, *p* = 0.002]. We also looked at age group differences in their feelings toward the charity foundation assessed by the additional questions we asked about the charity foundation. While the degree to which they agreed with the goal of the foundation did not differ across the two age groups [*t*_(175)_ = −0.34, *p* = 0.737], the degree to which the goal/mission of the charity affected their choice behavior was significantly higher in children/adolescents (3.49 ± 1.46) than in adults (2.79 ± 1.36) [*t*_(175)_ = −3.28, *p* = 0.001]. These results demonstrate that the chosen charity foundation (i.e., “Make a Wish!” foundation) was equally appealing to both age groups, although children/adolescents might have considered the goal/mission of the foundation to a greater degree when making their choices. When the happiness ratings to self and charity reward outcomes were compared across age groups using repeated measure ANOVA with domain (self vs. charity) as a within subject factor and age group as a between subject factor, we found a significant main effect of domain [*F*_(1, 175)_ = 19.64, *p* < 0.0001] and age group by domain interaction [*F*_(1, 175)_ = 47.95, *p* < 0.0001] (Figure 4). A paired *t*-test showed that while adults felt happier when winning money for themselves [*t*_(101)_ = 2.03, *p* = 0.045], children and adolescents felt happier when winning money for charity [*t*_(74)_ = −6.97, *p* < 0.0001].

We also examined correlation between age and these measures within the children/adolescents sample to look at age-related changes in other regarding behavior. A significant relationship (with Bonferroni correction for six different correlations, *p* < 0.05/6) was only found with the degree to which the goal/mission of the charity affected their choice behavior in the positive direction (*r* = 0.328, *p* = 0.004), suggesting that the degree to which the goal/mission of the charity influenced decisions increased with age.

## Discussion

We examined how reward learning unfolds in children and adolescents when decisions have consequences for both oneself and others. We used the previously developed social gambling task (SGT, Kwak et al., 2014), which captures tradeoffs between benefits to oneself and benefits to others under a dynamic environment that involves uncertainty. In this task, preferences for decks emerge over the course of the task unlike conventional single-shot games; this provides information about implicit learning of both non-social and social reward contingencies.

Our results from choices, decision strategies and the reinforcement-learning model collectively show that children and adolescents have greater sensitivity to rewards for others than for self. This was most notably found in the use of particular decision strategies. Specifically, the WL decision strategy—the proportion of same choices with the previous trial when the outcome was self win and charity lose—was used less often in children and adolescents as compared to adults. The WL strategy also negatively correlated with greater prosocial reward learning (i.e., greater value of LI_charity_–LI_self_). This implies that the younger group is more influenced by the charity outcomes than adults. Furthermore, the difference in reinforcement learning model parameter α showed that children and adolescents weigh charity reward relatively more than self reward compared to adults when updating their choices on a trial by trial basis. The results from SGT performance are also in line with the self-report measures of other-regarding behavior as shown by higher altruism score and relatively higher happiness ratings when winning money for charity than self in children and adolescents as compared to adults.

The use of a reinforcement learning model to explain the reward learning process in the two age groups provides new insight into the development of pro-social decision making. Specifically the parameters of the reinforcement learning model reveal trial-by-trial behavioral adjustments following the reward outcomes for self and charity. Our results demonstrate that children/adolescents differ from adults in how they incorporate self and charity rewards across trials, a finding not necessarily evident from the aggregate choices.

It is of note that the choice behavior in this task was more variable in the younger group than adults, as reflected in several results. First, children and adolescents did not show an equivalent level of learning as the adults as shown by slower changes in LIs across blocks. This suggests that they did not consistently choose the decks associated with overall gain and switched their choices more so than adults. This pattern is more apparent when looking at the decision strategy measure, which gives the degree to which the same card was selected across two consecutive trials. Children and adolescents showed an overall reduction in the use of these strategies suggesting that they switch between card decks more than adults. Lastly, the fact that model performance was worse in the younger group also suggests greater variability in choices. Heightened behavioral variability in children as compared to adults is a widely accepted finding that has been suggested to be a central feature of cognitive development (Elliott, 1970; Siegler, 1994; Williams et al., 2005; Tamnes et al., 2012).

Several limitations of the current study provide directions for future work. First, some participants (in both age groups) failed to choose the positive reward decks consistently (i.e., the No Knowledge groups). This may or may not reflect an absence of successful learning. It remains possible, for example, that some participants do correctly identify the “good” and “bad” decks but do not consistently avoid the “bad” decks or seek the “good” decks. This could occur because of a bias toward sampling new decks, because of a lack of utility associated with a given deck (e.g., not supporting the charity), or for some other reason. Second, we used a wide age-range of children and adolescents within our sample. Given the recognized developmental changes in decision making from childhood to adolescence (Crone and Dahl, 2012)—and the correlations with age we observed in the present study—future research should narrow down the timing of developmental transitions within this large range. Third, the pro-social behavior we observed in this study could have been influenced by preferences specific to the chosen charitable foundation (“Make a Wish!”). This charity was selected to be appealing to both our younger and older participants, and indeed our two age groups exhibited similar levels of agreement with its goal and mission. However, we cannot completely rule out the possibility that a different pattern of result could be observed with a different social target.

Change in sensitivity to social contexts across development is a widely studied topic (Nelson et al., 2005; Blakemore, 2010; Burnett et al., 2011; Crone and Dahl, 2012). Development of social cognitive skills including mentalizing and perspective taking during adolescence allows increase in prosocial behaviors as supported by self-report measures of altruism (Eisenberg et al., 1995, 2002, 2005; Eiesnberg and Fabes, 1998; Killen and Turiel, 1998; Carlo et al., 1999, 2007; Fabes et al., 1999; Wentzel et al., 2007; Luengo Kanacri et al., 2013, 2014). These characteristic developmental changes in mental representations of choices, reward sensitivity and self-control abilities have been invoked to explain greater sensitivity to rewards and increased tendency to take risks (Casey, 2015; Reyna et al., 2015). Our finding of heightened sensitivity to social rewards in children and adolescents provides a clear, well-controlled test of how the decision-making of pre-adolescents and adolescents changes under a social environment in which others' rewards are also at stake. These results are also consistent with recent findings showing greater risk taking in adolescents when surrounded by peers (Albert et al., 2013).

SGT was modeled after the Iowa gambling task (IGT), which has been repeatedly used in children and adolescent populations (Lehto and Elorinne, 2003; Hooper et al., 2004; Crone and van der Molen, 2007; Crone et al., 2008; Cauffman et al., 2010; Smith et al., 2012); thus, the SGT may also become a viable tool for this younger population. While some of these studies showed age related increase in performance across adolescence to adulthood (Hooper et al., 2004; Crone and van der Molen, 2007; Cauffman et al., 2010), there have also been reports of mid-adolescence specific impairment showing a curvilinear trend across development (Lehto and Elorinne, 2003; Smith et al., 2012). Based on these previous reports, one might question whether the age group differences in social reward learning observed in SGT reflect age-related changes in general cognitive abilities instead of changes in social preference. To address this issue, we also ran all of our analysis after excluding individuals in the No Knowledge subgroup in both adults and children/adolescents (i.e., those who presented no evidence of understanding of the task; see Supplementary Materials). This allows us to limit our analysis within individuals demonstrating the capability to sufficiently perform the task. Within this subset of participants, we actually found a significant age group by domain (i.e., self vs. charity) interaction in mean LI. This suggests that age group differences in reward learning across the self and charity domains is not necessarily a result of the difference in general cognitive abilities between the age groups.

Another possible interpretation of our data is that children and adolescents are trying to signal personal altruism—a desirable trait in our society—instead of actually having greater social preference. The possibility that the children and adolescents give more socially desirable responses has similarly been noted in prior research using self-report measures (Eisenberg et al., 1995, 2002, 2005; Eiesnberg and Fabes, 1998; Killen and Turiel, 1998; Carlo et al., 1999, 2007; Fabes et al., 1999; Wentzel et al., 2007; Luengo Kanacri et al., 2013, 2014). However, our task paradigm provides a measure of *implicit* sensitivity toward rewards for others. Specifically the reinforcement learning parameter α describes trial-by-trial adjustments in behavior based on their sensitivity to the reward outcome for charity vs. self. This implicit measure of prosocial tendencies would be more resistant to social desirability effects than would traditional explicit measures (e.g., surveys).

The current study investigated how children and adolescents process rewards directed to self and others by using the Social Gambling Task (SGT), a paradigm that allows measuring the dynamics of reward learning for oneself and for a socially desirable target. Our results suggest that children and adolescents have increased sensitivity to social outcomes that in turn leads to procial behaviors such as donating to charity. These results provide new insight into the contributions of social information to decision making and have broader implications for understanding the development of social decision making.

## Author contributions

YK and SH conceived and designed the experiments. YK performed the experiments. YK and SH analyzed the data and wrote the paper.

### Conflict of interest statement

The authors declare that the research was conducted in the absence of any commercial or financial relationships that could be construed as a potential conflict of interest.
