# Supplementary material for: Prosocial Reward Learning in Children and Adolescents
Source: Front Psychol. 2016 Oct 5;7:1539. doi: 10.3389/fpsyg.2016.01539 (PMC5050220; doi:10.3389/fpsyg.2016.01539)
Supplement: Supplementary file 1 [file DataSheet1.DOCX]

***Supplementary Material***

Prosocial reward learning in children and adolescents

**Youngbin Kwak & Scott A. Huettel**

*** Correspondence:** Scott Huettel: scott.huettel@duke.edu

**Social Gambling Task (SGT) instructions**

We gave the following instructions to the participants: “In this study you will earn money for yourself and for a charity by playing a card deck game. There will be 4 card decks shown on the screen horizontally. You will select one card at a time by pressing the corresponding four keys. Each card draw will tell you how much you won for yourself and for the charity. For both domains, sometimes you will win more money than others and sometimes you will even lose money. The total money you earned for yourself and for charity will be shown on top of the screen. You are absolutely free to switch from one deck to another at any time, and as often as you wish. The only hint we can give you, and the most important thing to note is this: Out of the four decks of cards, there are some that are worse than others for you or for charity. Also note that the computer does not change the order of the cards once you begin the game. That is, it does not make you win or lose at random.”

**Survey questionnaires**

The HOQ questionnaire comprises twenty-three multiple-choice questions, such as: *“A person in one of your classes is having trouble at home and with school work. You: A) help the person as much as you can. B) tell the person not to bother you.  C) leave the person alone to work out his or her own problems. D) agree to tutor the person for a reasonable fee”.* Experimental studies show that greater altruistic tendencies as measured by HOQ predicted greater helping of a person in need ([Carlo, Eisenberg et al. 1991](#_ENREF_10)). The HOQ was not particularly designed for adolescent population however we included this measure based on our previous finding of showing association with pro-social reward learning in adults ([Kwak, Pearson et al. 2014](#_ENREF_34)).

We also asked the participants the following questions related to the charity foundation: 1) How much do you agree with the goal of this charity foundation? 2) How much did the goal/mission of the charity affect your decision in choosing the card decks? 3) How did you feel when you won money for yourself? 4) How did you feel when you won money for the charity? Participants gave responses in a five-point Likert scale to each of the questions. The HOQ was collected in 60 out of 75 adolescents and the happiness ratings for self and charity was collected in 73 out of 75 adolescents.

**SGT performance of adolescents and adults after excluding No Knowledge group**

A repeated measures ANOVA with domain (CI_self_ vs. CI_charity_) by block (block 1-10) as within-subject factors and age group (children/adolescents vs. adults) as a between-subject factor was performed to determine how learning emerged across time. We found a significant main effect of block (F_6.43,597.93_ = 22.83, p < 0.0001) and age group by domain (F_1,93_ = 5.83, p = 0.018) and age group by block (F_6.43,579.93_ = 3.06, p = 0.005) interaction. A follow up paired t-test comparing block average CI of self and charity in each age group showed a significantly greater average CI in self than charity in adults (t_59_ = 2.31, p = 0.024, self (*M* ± *SD*): 4.96 ± 2.89, charity: 3.74 ± 3.69), but no significant difference in children/adolescents (t_34_ = -1.45, p = 0.16, self: 3.31 ± 2.62, charity: 3.95 ± 2.78).

Next, we compared the use of the four strategies (i.e. same choice of card deck as the previous trial when the outcome of the previous trial was self win and charity win (WW), self win and charity lose (WL), self lose and charity win (LW), and self lose and charity lose (LL).) across age groups using a repeated measures ANOVA with age group as a between subject factor and strategy type as a within subject factor. We found a main effect of strategy type (F_3,279_ = 53.79, p < 0.0001), age group (F_1,93_ = 32.72, p < 0.0001) and age-group-by-strategy-type interaction (F_3,279_ = 4.86, p = 0.003). Children/adolescents were using these strategies less than adults. Visual inspection of the data showed that the two age groups differed the most in the “WL” strategy as was found in previous analyses including data from all study participants. To test for this, we ran the same ANOVA only including the other three strategies. Age group by strategy type interaction was no longer present (F_2,186_ = 0.023, p = 0.97) suggesting that the previously found interaction effect was driven by “WL” strategy.

**Supplementary Figure 1.** Correlation between age and choice index in self domain (left) and between age and WW (self win and charity win) strategy use (right) within children and adolescents.
